# Clinicians’ and patients’ experiences of managing heart failure during the COVID-19 pandemic: a qualitative study

**DOI:** 10.3399/BJGPO.2021.0115

**Published:** 2021-10-13

**Authors:** Faye Forsyth, Emma Sowden, Muhammad Zakir Hossain, Rachel Tuffnell, Carolyn Chew-Graham, Thomas Blakeman, Christi Deaton

**Affiliations:** 1 Clinical Nursing Research Group, Primary Care Unit, Department of Public Health & Primary Care, University of Cambridge, Cambridge, UK; 2 Division of Population Health, Health Services Research and Primary Care, School of Health Sciences, University of Manchester, Manchester, UK; 3 Public Health, Policy, and Systems, Institute of Population Health Sciences, University of Liverpool, Liverpool, UK; 4 School of Medicine, Faculty of Medicine and Health Sciences, Keele University, Keele, UK

**Keywords:** qualitative research, COVID-19, heart failure

## Abstract

**Background:**

Severe acute respiratory coronavirus 2 (SARS-CoV-2), also known as coronavirus disease 2019 (COVID-19), resulted in unprecedented societal and healthcare provision change, which has been implemented at pace. Little is known about the indirect impacts of these changes and what the future effects may be.

**Aim:**

To explore patients’ and clinicians’ experiences of managing heart failure (HF) during the COVID-19 pandemic.

**Design & setting:**

Qualitative study in three regions of the UK: Cambridgeshire, Greater Manchester, and the West Midlands.

**Method:**

Semi-structured interviews (*n* = 30) were conducted with older adults with established HF and healthcare providers from primary and secondary health services involved in their care. Interviews were analysed thematically.

**Results:**

Compliance with the government guidance ‘Stay at home, protect the NHS, and save lives’ during the COVID-19 pandemic, and perceptions relating to risk from COVID-19 and underlying morbidity, drove ‘being careful’ behaviours and organisational changes. Enacting behavioural change and implementing organisational change resulted in opportunities and challenges for health and healthcare practice.

**Conclusion:**

Perception of risk led to significant behavioural and organisational change during the pandemic. Some changes described by both patients and clinicians, such as enhanced relationships and self-monitoring, present as opportunities, and consideration should be given as to how to maintain or develop these. Equally, indirect impacts of COVID-19 and the associated lockdown, such as disengagement and withdrawal, and the fallout from reluctance to access health services, should be acknowledged and interventions to address these challenges are needed.

## How this fits in

This study describes how the fear of contracting COVID-19 in people with HF appeared to influence behaviour, with both health-promoting and health-avoidance responses reported by both patients and clinicians. Some behavioural responses present as opportunities for health improvement, while others may require focused interventions to limit possible detrimental effects.

## Introduction

Numerous studies have shown that healthcare services involved in the prevention and treatment of non-communicable diseases in the UK have been severely disrupted since the onset of COVID-19.^1^ Some of these report that HF services have been disproportionately affected as staff and services were reorganised to support the surge of COVID-19 admissions,^2^ or to manage the substantial cardiovascular sequelae.^3^ Retrospective analysis of routine hospital data demonstrates the extent to which healthcare utilisation decreased compared with activity from previous years and confirm recovery has been limited by successive waves of infection.^4^ Analysis in primary care reveals consultation rates reduced dramatically in the acute phase of the pandemic, remote consulting became the norm, and practitioners focused on older, vulnerable patients or those with poor mental health.^5^

These statistics are concerning given that: 1) older adults with HF were not officially recognised on the extremely clinically vulnerable shielding list (a list of vulnerable patients identified by NHS England, who were thought to be at high risk of complications from COVID-19, and were subsequently sent a letter with advice on how to protect themselves); 2) patients with HF have an increased risk of poor outcomes like chronic disability, regardless of a pandemic;^6,7^ and 3) patients with HF have a significant risk of severe illness and death with concomitant COVID-19 infection, or rapid deterioration owing to cancellation of services and disbanded routine monitoring and prescribing.^2,8^

The purpose of this research was to explore patients’ and providers’ experiences of managing established HF during the COVID-19 pandemic. Research questions focused on experiences and actions taken during the pandemic, changes in care practices, and consequences of changes. Findings are reported in line with The Standards for Reporting Qualitative Research.^9^

## Method

### Approach

Qualitative semi-structured interviews were used. This allowed for broad inclusion, exploration, and description. The research team consisted of experienced health services researchers, including practising clinicians (CC-G, TB, RT). Interviews were conducted by three researchers (FF, MH, ES) who had established rapport with the interviewees through prior studies.^10–12^ The same researchers familiarised themselves with, categorised, and collated data into descriptive codes in an inductive approach; 50% of all interviews were double-coded. All authors contributed to searching, reviewing, and defining themes. Sensitising concepts were selected iteratively and used deductively in the reviewing and defining phase to inform theme definition and narrative, to theorise, and to aid interpretation of the data.

### Sampling strategy

**Figure 1.**
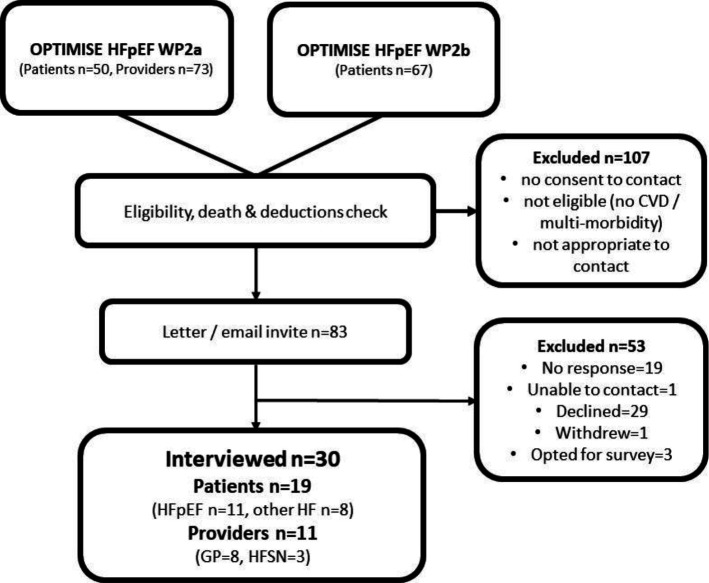
Participant recruitment flow diagram. CVD = cardiovascular disease. HF = heart failure. HFSN = heart failure specialist nurse. OPTIMISE HFpEF = Optimising Management of Heart Failure with Preserved Ejection Fraction in Primary Care. WP = work package.

Participants were recruited from two cohorts from Cambridgeshire, Greater Manchester, and the West Midlands, which had been established as part of a programme of research.^10^ Inclusion criteria were deliberately broad; only those who did not have an extensive HF history, those who were not thought to be appropriate to contact, and those without consent to be recontacted were excluded. Healthcare providers involved in the care pathway of people with HF were also recruited. Eighty-three participants (46 patients and 37 clinicians) out of a possible sample of 190 were eligible to participate. Recruitment was terminated when the maximum number of invites set by ethical approvals were reached. Figure 1 demonstrates the processes of recruitment, response rate, and eventual sample.

### Ethical issues

Substantial amendments to existing studies were submitted to allow recall of participants. All participants provided written informed consent, and interviews were stored securely according to data protection regulations and site polices. Transcripts were fully anonymised and kept separate from identifiable data.

### Data collection

Potential participants were provided study information, a response pro forma, and a consent form. In total, 30 interviews were conducted with a mean length of 48 minutes. Nineteen interviews were with patients (denoted with participant codes beginning with ‘P’) and 11 with healthcare providers (participant codes beginning ‘HCP’). Of the patients, 11 had a diagnosis of HF with preserved ejection fraction (HFpEF). The median age of patients was 80 years and all were shielding in some form (Table 1). Within the provider group eight were GPs and three were HF specialist nurses. Participants were recruited and interviewed between May and October 2020; between national lockdowns 1 and 2, a period of progressive easing of restrictions.

**Table 1.** Participant characteristics

**Patient characteristics (*n* =** **19)**	**Provider characteristics (*n* =** **11)**
Age range, years	65–92	Female sex, *n* (%)	7 (64)
Female sex, *n* (%)	7 (37)	GP, *n* (%)	8 (73)
HFpEF, *n* (%)	11 (58)	Heart failurespecialist nurse, *n* (%)	3 (27)
Other HF, *n* (%)	8 (42)		

HF = heart failure. HFpEF = heart failure with preserved ejection fraction.

Interviews were conducted by phone or video-conferencing given COVID-19 restrictions. Interviews were semi-structured and guided by their respective topic guide (for healthcare professionals see Supplementary materials Appendix S1; for patients, Appendix S2). Topic guides were not changed, but subsequent interviews were informed by the analytic process.

### Data analysis

Recordings were transcribed verbatim and managed with NVivo 12 software. An inductive and deductive approach, following the six phases as outlined by Braun and Clarke, was undertaken.^13^ Initial codes describing the content were applied and an accompanying analytic notebook was circulated, documenting evolving relationships and understanding of codes, and the influence of context, situation, and the researchers’ interpretations. Virtual analytic meetings were held once every 2 weeks, wherein codes were discussed by the team and assembled or disassembled into potential themes. Potential themes were visualised in thematic maps that were discussed and sense-checked for internal (code level) and external (data corpus level) coherence. Thematic maps were continually reviewed and refined as data-driven sensitising concepts from the literature were introduced and overlaid.

## Results

Compliance with government lockdown guidance ‘Stay at home, protect the NHS, and save lives’ and perceptions relating to risk (theme 1) drove ‘being careful’ behaviours (theme 2) and organisational change to provide safe systems of care (theme 3). Enacting behavioural change and reorganisation of care has resulted in opportunities (theme 4) and challenges (theme 5) for health care.

### Theme 1: Balancing risks and risk uncertainty

Risk was described by both patients and providers in three sub-themes. Risk associated with contracting COVID-19, the risk of deteriorating from underlying conditions like HF and/or comorbid conditions, or in relation to uncertainty regarding risk judgments. Concern about contracting COVID-19 when older and living with HF was universally acknowledged and heavily influenced behavioural responses to the pandemic. Those who appraised the personal threat of infection as significant implemented the most robust protective measures:

**‘*I’m male, which is a problem, I’m over 60, which is a problem, I’m type 2* *diabetic, which is a problem, I’ve got an underlying heart condition, is a problem, and they would class me as obese, which is a problem. So I hit a lot of the at-risk buttons. And my* [wife] *has got asthma as well so we’ve tended to assume that we need to be careful.* *’* (P001)

**‘[*…] a lot of the patients are very anxious about leaving their home or being seen anywhere or being examined because they didn’t want to risk catching the virus.* *’* (HCP07)

While no patients described a deterioration in their HF status, some did reflect on balancing the risk of virus exposure versus the risk of deterioration from underlying conditions. Balancing these competing risks was frequently at the forefront of decisionmaking for many clinicians managing patients whose conditions were deteriorating:

*‘* *So my threshold for sending people into hospital with long-term conditions was raised because I had to balance the risk of problems from the long-term condition against the risk of what would happen if they caught coronavirus while they were in hospital.* *’* (HCP07)

*‘* *So that there is a risk that I get Covid, there’s a certainty if I stop walking I’d have a problem.* *’* (P001)

Both groups discussed the challenge of gauging clinical risk and there was significant uncertainty, particularly in the absence of specific shielding guidance for people with HF. Clinicians reported uncertainty over risk stratification, especially when health status was not easily elicited via remote consultation or in relation to government advice on suspension of routine services:

**‘*But with heart failure, it’s very visual … I can tell a lot from how somebody gets up in the waiting room and walks into the clinic room how they're coping with their breathing and their mobility … we're dependent really on what they can tell us on the phone, how savvy they are at communicating.* *’* (HCP10)

**‘*If somebody had sent me a letter and said “you’re shielding now for 12 weeks”, I can accept that, I can do it. But if nobody tells me, why should I do it? I don’t pose a risk or am I expendable? I don’t know.* *’* (P008)

### Theme 2: ‘Being careful’ behavioural responses

A variety of behavioural responses, driven by government mandate but also perceptions of risk, were reported and categorised under three sub-themes: engaging support networks, self-monitoring, and enhanced personal protective strategies. Patients typically described responses as ‘being careful’ or ‘sensible’. They attempted to mitigate risk of contracting COVID-19 by engaging support networks (family, community, health services) to limit exposure. Clinicians similarly spoke of engaging or utilising support networks (teamwork, collaboration) to maintain patient safety:

**‘*I was having more conversations with specialists themselves on the telephone which I never used to do to try and manage patients who were shielding. So, between us, we could manage the patient without them having to come in twice to see me and to see the specialist.* *’* (HCP08)

Personal risk avoidance strategies were variable and depended on perceived risk of infection from either physical contact, populated environments, and fomites (such as food and mail). Those perceiving the greatest risk enacted the most stringent protective behaviours. Clinicians who were more concerned about contracting and spreading the virus to vulnerable patients, family members, or colleagues often implemented personal strategies not mandated at the practice level.

**‘[*…] things that are delivered by post, depending on what they are, paper stuff sits aside for sort of about twenty-four, thirty hours before we open it.*‘ (P004)

Both patients and clinicians reported increased efforts in self-monitoring conditions when equipment and ability allowed. Both attributed this change to avoidance of physical attendance at healthcare settings:

**‘*There’s been quite a shift in the patients with long-term conditions. A lot of them have got hold of devices to monitor themselves […] So they have shifted towards self-monitoring and then providing us with that information more than they ever would have done before.* *’* (HCP08)

### Theme 3: Organisational responses — providing safe services

To reduce transmission, NHS England and NHS Improvement issued COVID-19 operational standards^14^ that recommended total digital triage, reconfiguration of physical spaces, and prioritisation of non-emergency care.^15^ In some settings, reconfiguration expedited planned restructuring; for others it represented greater adjustment. The main methods reported were remote triage, multi-modal remote consultation (telephone, video, online), and COVID-19 secure face-to-face consultations. With the exception of convenience and efficiency, perceptions of new operating models were varied and influenced by clinical risk and the service users’ technological capability, similar to that described in other studies:^16^

**‘*The video normally supplements the phone consultations. So normally it would be phone first and then video if it’s necessary to add something or if it’s a patient request … one thing that has dramatically changed is our use of text messaging … and it’s even possible to do some long-term condition reviews by SMS.* *’* (HCP01)

**‘[*…] only a couple of members of the team have used* [video-conferencing] *yet, they found it very time-consuming and challenging with getting the software to work with patients* *.’* (HCP10)

### Theme 4: Opportunities post-COVID-19

Some behavioural and organisational responses present as opportunities to cement health and care change. For example, engaging networks has strengthened personal social networks of support, and efforts to avoid healthcare settings has led to greater adoption of self-care and proactive health behaviour change. Aspects of organisational change were perceived to be more convenient, efficient, and responsive. Patient participants reported accessing community initiatives and utilising local support in the form of neighbours and friends. There was also testimony that this cohesion persisted beyond the formal lockdown period. Clinicians described greater collaboration with colleagues internally and across organisational boundaries.

**‘*We’re very lucky that the people who have helped, my youngest son doesn’t live far away and he goes shopping for us, and the young lady over the road came and introduced herself, quite a new neighbour, and she’s been doing shopping for us. And our next door neighbour, the husband is shielding, so they get a priority slot at* [supermarket]*, so they get stuff for us as well.* *’* (P002)

In addition to an uptake in self-monitoring, some participants engaged in positive behaviour change as a means to avoid deteriorations in heath and reduce risk of poor outcome if they contracted COVID-19:

**‘[...]* before* [lockdown] *I didn’t necessarily go out* [walking] *every day. But once it was rationed, you know what it’s like, once it’s rationed you like to get your share.* *’* (P004)

Although not all organisational changes were universally perceived to be positive, benefits were observed. The convenience and efficiency of telephone consultations were noted, saving patients time and money. However, in almost all cases, the appropriateness of non-face-to-face consultations was considered situation dependent and there was concern over the ‘digital divide’:^17^

**‘*But imagine, forget lockdown, imagine how long it would have taken me to have the same 10* *-minute* *conversation* [about a routine echocardiogram]*. It would have taken me the thick end of a day to go and visit. Would I have got any more information? Well I don’t think I would.* *’* (P001)

### Theme 5: Challenges post-COVID-19

Patients in this cohort did not report a deterioration in their HF that necessitated accessing emergency or specialist services; however, they reported hypothetical reluctance and refusal to access healthcare settings if faced with a HF deterioration. Many patients reported cessation of leisure activities previously performed owing to lockdown measures or perceived risk, which were not reinstated once permitted. The potential for diminished resilience, the process of adapting to adversity, also featured for both patients and providers.

Reluctance and refusal appeared to be influenced by a number of factors, including healthcare settings as a source of infection, perceptions regarding rationing and prioritising in a system that was reportedly overrun, and in terms of altruism or responsible use of services. One patient reporting an emergency health event unrelated to HF (spinal fractures) declined to attend accident and emergency (A&E) based on their perception of the risks associated with hospitalisation:

**‘*Stay away from hospitals* *...* *that was a, a rotten time i* *n ...* *this flipping pandemic.* **“*But the doctors need to see you.* *”* *I said,* *“* *Look, I'm the wrong age. … you want to get me down these stairs, into an A&E which, in everyone’s own admission, is not a safe place to be anymore. So I could go in* *...* *looking for potential breaks in the back, or whatever it might be, but I might not come out of it …* *”* **“*Oh well, that is a risk.*”** *I said,* **“*Well, that’s a risk I'm not prepared to take.* *”* *’* (P019)

**‘*But if you listen to the news, if I’d have gone to the hospital for anything I would have picked COVID up* [*…]* *I’d have been dead within the fortnight or on a life support machine. So the media puts this message out to me. If my heart would have gone, no, I wouldn't have gone to hospital.* *’* (P008)

A significant proportion of patients who ceased activities also reported delaying or not reengaging in usual activities, or voluntarily extending lockdowns. Providers were particularly concerned about the implications of withdrawal and isolation, predicting heavy psychosocial and physical tolls.

**‘*We were very, very active, we played golf a couple of times a week and we swam three times a week, so we were pretty active every day. Now, of course, this has all stopped since the swimming club closed down and the golf club closed down for a time, and we haven’t picked anything up.* *’* (P011)

Ceasing and delaying reengagement with pre-lockdown activities was linked to loss of confidence and diminished resilience, particularly in frailer participants reporting pre-pandemic functional impairment. For staff there were concerns over personal and professional resilience.

**‘*I just don’t want to go out. I just don’t feel safe because I mean he* [prime minister] *did say “don’t go on public transport” and I thought well I can get a lift if I wanted to* *…* *but I just don’t want to go.* *’* (P003)

**‘*You know, people haven’t had their holiday, so the breaks they need, and have had to work harder in more uncertainty than they have done before. And that’s not gone anywhere, that’s still there. But now the emphasis is on finding new ways to work and changing the system, when people have very little resilience.* *’* (HCP05)

## Discussion

### Summary

This qualitative article illuminates key themes for consideration in terms of redesigning services in light of the pandemic. The ‘Stay at home, protect the NHS, and save lives’ message disseminated by government at the height of the pandemic, alongside perceptions of risk from COVID-19, resulted in behavioural and organisational change intended to mitigate risks. While many of these changes are likely transient, some will have lasting impacts that require careful consideration and system change to either encourage maintenance or limit further adverse consequences.

### Strengths and limitations

To the authors’ knowledge, this is the first UK-based interview study that captures the views of patients with HF and the clinicians who cared for them during the pandemic. However, this study is limited by its retrospective nature, with participants being asked to recall their past experience of lockdown 1. Interviews were not longitudinal, therefore any changes in perspectives, as has been found in other studies,^5,18^ were not captured. All patient participants reported clinical stability in terms of their HF, therefore their accounts may be different from patients with HF experiencing a deterioration and unplanned hospitalisation. Demographic data collected was limited, therefore the influence of factors like socioeconomic status, which have been shown to affect outcome in HF,^19^ could not be accounted for. Lastly, recruitment of providers from the wider HF care team (cardiologists, rehabilitation specialists) was challenging due to workload, limiting this study’s representation of their perspectives on care.

### Comparison with existing literature

Five previous studies have focused on perspectives or responses of patients with HF to the pandemic. A large survey of 1050 patients with HF in the UK found that there was significant anxiety regarding exposure to infection, disruption to HF services, and medication prescription, and a third of patients (32%) were reluctant to attend hospital.^2^ A survey of 109 patients with HF in the US, reported that most patients were worried about infection; however, levels of hesitancy towards accessing health care were lower.^20^ Chagué *et al* interviewed 124 patients with chronic HF and reported increased psychological distress and unhealthy lifestyle behaviours.^21^ An interpretative phenomenological analysis of interviews with 14 patients with HF described three themes: vulnerability, uncertainty, and positive coping strategies that typified patients’ experiences.^22^ A qualitative exploration of the impact of COVID-19 on self-care behaviours in older adults with HF in Texas, US, established that during COVID-19 there were multiple threats (social isolation, disruption to services) and safeguards (health-promoting activities and connectedness) to sustaining self-care behaviours, particularly physical activity.^23^

Consistent with these studies, the present study found that patients with HF perceived great risk from infection, which influenced behaviours including healthcare avoidance. In contrast to earlier studies describing unhealthy lifestyle behaviours, it was observed in the present study that many patients with HF made or experienced positive health-protective changes. While most large-scale studies have documented trends towards worsening health behaviours during lockdown,^24–26^ the present study is not alone in describing divergence. Within their analysis of experiences of patients with HF, both Trenta *et al* ^22^ and Radhakrishnan *et al* ^23^ describe positive lifestyle and social connectedness changes made by patients. In non-HF populations, a large study of pre-peri-post-pandemic diet and health behaviours in 1.1 million UK and US individuals similarly suggest that the pandemic may have provided impetus to improve health behaviours.^27^ A survey of the food habits of 240 UK adults also reported the majority of their sample placed more importance on health and weight control during lockdown.^28^

There remain relatively few UK studies on the experiential impact of the pandemic on primary care. A qualitative interview study of 132 GPs conducted in Flanders reported that, while practitioners rapidly and successfully adjusted to pandemic guidance, they were concerned about: contracting the virus and becoming a source of infection; the continuity of regular care; and consequences of anti-COVID-19 measures.^29^ Another Belgian study (*n* = 21) also reported severe disruption to routine care, large-scale reorganisation, and the challenges of risk stratification.^30^ An Italian study described how lack of organisation and cooperation across institutions induced a sense of abandonment; however, embracing digital technologies and local networks helped practitioners to cope with change.^31^ A rapid review of the effects of COVID-19 on the mental health of healthcare workers found significant impacts on the psychological wellbeing of providers, which was potentially mediated by resilience.^32^ Lastly, a survey of 257 GPs in Singapore reported that anxiety, burnout, and depression were more highly prevalent compared to pre-COVID-19, with changes to practices, increased workloads, and financial difficulties being highlighted as contributors.^33^

There are substantially more reports quantifying pandemic-related organisational change and the impacts on healthcare utilisation. A patient-level data analysis confirms consultation rates fell, were most often remote, and continued to be so post-lockdown.^34^ A systematic review of consultation rates concluded healthcare utilisation decreased by one-third during the pandemic.^35^ Ball and colleagues' analysis of hospital activity data reveal there was substantial reduction in total cardiovascular activities with limited recovery towards pre-pandemic levels.^4^ Murphy and colleagues' mixed-methods examination of changes in consultations during the pandemic found that telephone consultation rose significantly and while clinicians considered this an achievement, as time progressed, remote methods were described as mentally intense, straining, and less satisfying.^5^ Finally, a survey and consensus-building study in clinicians and patients with chronic obstructive pulmonary disease (COPD) found that clinicians saw many benefits to remote approaches, including the possibility of prompt, flexible, and ongoing contact with patients.^36^

In relation to the latter research, the present study's findings are similar; clinicians worked hard to implement safe services through embracing remote methods and utilising networks in the context of clinical prioritisation. Some components of this enforced change were perceived to be positive; however, there is concern about the indirect effects of the pandemic and continued personal and professional resilience.

### Implications for research and practice

Despite similarities with extant research, novel insights are described, particularly in relation to perceptions of risk and the downstream effects. Risk perceptions have been shown to correlate with multiple socio-cultural and experiential factors and are strongly associated with pro-social, altruistic world views.^37^ Appealing to these characteristics — as the UK government's ‘Protect the NHS’ slogan did — is likely to have contributed to healthcare avoidance behaviours reported here. Reluctance in help-seeking beyond lockdowns may persist, particularly if reports of extreme pressure and backlogs in elective care continue. It is important that further research explores perceptions and evolution of risk and the impact on health-seeking behaviour in order to ensure appropriate information on risk is updated and communicated.

This study highlighted the challenges of deconditioning, social withdrawal, and reduced resilience, which could lead to functional and mental health decline.^38^ Conversely, there were also reports of strengthened personal social networks, increased self-care, and the greater convenience of services, which represent opportunities for change. As planning and implementation of the NHS Long Term Plan^39^ is already underway, placing the study's findings within the context of this policy is critical. Central to delivering objectives in the NHS Long Term Plan is the implementation of integrated care systems (ICS) across England.^40^ Described as collaborations of local organisations, ICS will be responsible for planning and commissioning joined-up care around people and neighbourhoods, rather than institutions.

Participating in community assets has been shown to improve quality of life of, and reduced care costs in, older adults, and conversely ceasing engagement in community assets was associated with increased health problems and higher care costs.^41^ Given this, ICS that have already planned priorities, and those in the planning process should consider pandemic-related deconditioning and social withdrawal as potential priorities requiring community-based rehabilitation initiatives. Community assets and social prescribing could be bolstered to address these detrimental effects. Improved management of long-term conditions is likely to be an ambition of most ICS, given population trends. This research would suggest that COVID-19 has been a catalyst for greater proactive health and self-care; therefore, consideration should be given as to how to embed and encourage health vigilance through supportive services. While the pandemic has brought largely negatives impacts, it is important that the few positive effects are recognised and incorporated within ICS priorities.

In conclusion, perceptions of risk led to significant behavioural and organisational change during the pandemic. Some changes, such as greater self-monitoring, present as opportunities and consideration should be given as to how to maintain or develop these, particularly as these changes align with aims set out in the NHS Long Term Plan. Equally, negative impacts like disengagement and withdrawal, which were reported despite strengthened personal social networks of support, and the fallout from reluctance to access health services, should be acknowledged and interventions to address these challenges are needed.
